# Why Did You Choose This Pet?: Adopters and Pet Selection Preferences in Five Animal Shelters in the United States

**DOI:** 10.3390/ani2020144

**Published:** 2012-04-10

**Authors:** Emily Weiss, Katherine Miller, Heather Mohan-Gibbons, Carla Vela

**Affiliations:** 1 Shelter Research and Development, Community Outreach, American Society for the Prevention of Cruelty to Animals (ASPCA^®^), 424 E. 92nd St., New York, NY 10128, USA; 2 Applied Science and Research, Community Outreach, American Society for the Prevention of Cruelty to Animals (ASPCA^®^), 424 E. 92nd St., New York, NY 10128, USA; E-Mail: katherine.miller@aspca.org; 3 Applied Research and Behavior, Community Outreach, American Society for the Prevention of Cruelty to Animals (ASPCA^®^), 424 E. 92nd St., New York, NY 10128, USA; E-Mail: heather.mohan-gibbons@aspca.org; 4 Hunter College, City University of New York, 695 Park Ave., New York, NY 10065, USA; E-Mail: ci_vela@yahoo.com

**Keywords:** adopter, adoption, dog, cat, animal shelter, appearance, behavior, greeting, interaction, selection

## Abstract

**Simple Summary:**

This study examined reasons why adopters chose their pet in an animal shelter, what behaviors were first exhibited by the pet to the adopter, what information was important during their selection process, and the relative importance of seeing the animals’ behavior in various contexts.

**Abstract:**

Responses from an adopter survey (n = 1,491) determined reasons for pet selection, type of information received by the adopter, and the context in which the animal’s behavior was observed. Appearance of the animal, social behavior with adopter, and personality were the top reasons for adoption across species and age groups. Most adopters stated that information about the animal from a staff member or volunteer was more important than information on cage cards, and health and behavior information was particularly important. Adopters found greater importance in interacting with the animal rather than viewing it in its kennel. The results of this study can be used by shelters to create better adoption matches, prioritize shelter resources and staff training, and potentially increase adoptions. Additionally, some simple training techniques are suggested to facilitate adopter-friendly behaviors from sheltered dogs and cats.

## 1. Introduction

Approximately 1 million dogs and 2 million cats are adopted from animal shelters each year [1,2,3], meaning that about 23% of all owned dogs and 25% of owned cats were selected from shelters [1,4]. While there have been several studies investigating the change in the human-animal bond when an animal is relinquished to a shelter [5,6,7,8], there has been little inquiry or clear findings regarding how and why a particular animal is initially selected in a shelter.

Posage [9] reported that purebred dogs, small dogs, and dogs of certain coat colors were more likely adopted during their study period than dogs with other physical characteristics. However, a study in an animal shelter in Ireland found that breed, age, size and color were not significantly different amongst dogs that were and were not adopted during the study [10]. This difference may be due to methodological differences or simply the populations available in the shelters at the time of study. 

Temperament or personality may be influential to animal selection. Podbersek and Blackshaw [11] surveyed cat owners in Brisbane, Australia. Although no respondents had acquired their pet from an animal shelter, some acquired their pet as a “stray” (not as a gift). When asked why they initially selected their cat, the most commonly cited reasons were “personality” and “appearance”. Similarly, in a survey conducted in Belfast, “temperament” was listed as the most important characteristic when selecting a dog from a shelter [10]. Neither study, however, collected details regarding aspects of the animals’ personalities or behavior at the time of selection. In a small study of cat selection from an animal shelter, adopters indicated a preference for cats they perceived as friendly, playful, happy, relaxed, and not sad or fearful [12].

There is some indication that particular behaviors affect adoption choices. Fantuzzi [13] reported that shelter cats who were active attracted more attention from potential adopters. Another study found that length of time until adoption was positively correlated with latency to approach an unfamiliar person, suggesting that approach behavior may affect adopters’ cat selections [14]. Similarly, dogs at the front of their kennels may attract more adopter interest and preference than those at the back [10].

Adoption selections may also be affected by the information provided by the shelter about a dog or cat. For instance, simply being labeled as “stray” may cause animals to be less preferable when compared to those listed as “owner surrendered” [10,14].

A variety of factors therefore apparently influence adopters’ selection of cats or dogs. However, adopters have not been asked for detailed information about why they chose their pet, including aspects of the animal, the information received, and their experience while meeting potential adoptees.

This information could be used to increase adoptions, particularly for animals repeatedly passed over by adopters. While some characteristics such as breed, size, age, or color could not be changed, others factors, such as the animal’s behavior, the information provided by the shelter, and the context in which an animal is made available for viewing are subject to modification. For example, provision of short daily training sessions for shelter dogs, including teaching them to walk forward in the kennel when approached, can increase their probability of adoption compared to dogs without such training [15]. Furthermore, because the information given and opportunities for interaction vary from shelter to shelter, learning what information and experiences adopters find most influential could help shelters meet adopters’ needs and expectations while streamlining their efforts.

The goals of the present study were to (1) discover reasons adopters chose their animal, (2) identify animal behaviors that played a role in the selection process, (3) determine what avenues of information gathering were important during the selection process, (4) understand the relative importance of seeing the animals’ behavior in various contexts, and (5) distinguish what other information influenced adoption.

## 2. Methods

### 2.1. Survey

This survey was conducted January through May, 2011 in five organizations around the United States, two of which are open-admission shelters that perform animal control services for their municipalities: Hillsborough County Animal Services (Tampa, FL, USA) and Charleston Animal Society (Charleston, SC, USA) and three of which are limited intake, privately-funded animal shelters: Animal Rescue Foundation (Walnut Creek, CA, USA), Wisconsin Humane Society (Milwaukee, WI, USA), and the ASPCA^®^ adoption center (New York, NY, USA). In all of these shelters except the ASPCA^®^, potential adopters are free to walk around to view many or all of the dogs and cats available for adoption, and seek the assistance of a staff member or trained adoptions volunteer when they wish to meet particular animals. At the ASPCA^®^, adopters are escorted by an adoptions staff member or volunteer as they view and meet the animals.

All organizations provide at least basic demographic and medical information on a card on each animal’s cage, but some animals have additional information provided, such as regarding behavior, training, and information from a previous owner, depending on what information the shelter has been able to gather. All shelters have areas where adopters can meet the animals prior to making an adoption selection. All organizations except Hillsborough County Animal Services facilitate dog-dog introductions prior to adoption if a dog already resides in the adoptive home. 

**Figure 1:**
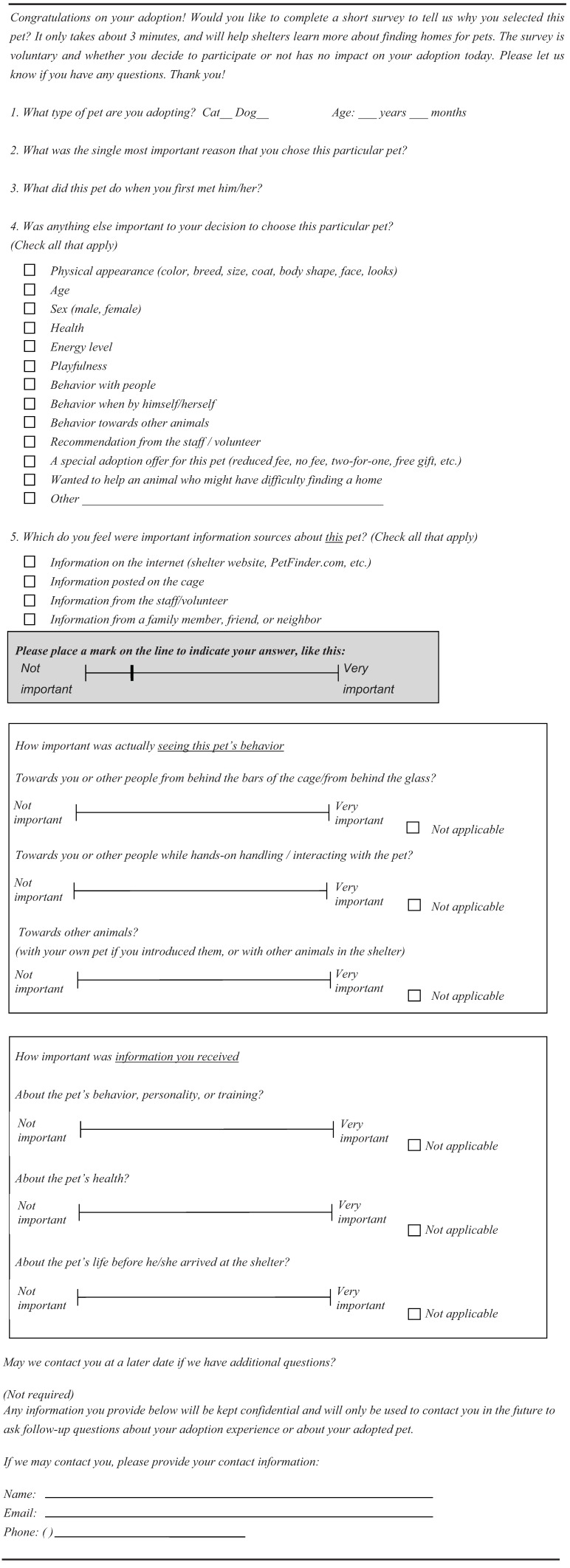
Adopter survey given to all five animal shelters.

The survey consisted of seven questions to determine reasons for pet selection, type of information received and the way it was received, and the context in which the animal’s behavior was observed (Figure 1). For the visual analog rating scales for questions 7 and 8, the respondent placed a mark on a line representing the range between two anchor points, “Not important” and “Very important.” The distance from the origin of the line to the participant’s mark served to indicate the degree to which the item was perceived as important. The actual length of the available line varied somewhat due to differences in the participating shelters’ printers, resulting in line lengths that varied across shelters from 6.5 cm to 8.0 cm. The survey was pilot tested for clarity on several ASPCA^®^ colleagues before finalizing the question format and then translated into Spanish by two native Spanish speakers. Immediately after adopters choose their pet, they were asked by a shelter staff or volunteer to complete the survey. Adopters were assured that filling out the survey was voluntary and that it would not affect their adoption. This information was also available in print at the top of the survey for adopters to read. They were offered the choice of completing the survey in English or Spanish. If a respondent was adopting more than one animal, he/she was asked to complete the survey for just one of the animals, of his/her choosing.

### 2.2. Data Analysis

Data were analyzed separately for kittens, cats, puppies and dogs. Kittens and puppies were defined as animals reported in Question 1 to be under five months of age [16], which can be readily determined by evidence of eruption of adult canine teeth in dogs and cats.

For the open-ended questions (Questions 2 and 3) the participant’s written responses were categorized for data analysis. Many responses pertained to behavior, therefore one of the authors (KAM) who is a Certified Applied Animal Behaviorist, directed response categorization. In order to retain as much detailed information as possible, the category “Personality/temperament” was only used when these terms were actually written by the respondent, and no other information was given about *type* of personality or temperament. Some respondents provided multiple answers for Questions 2 and 3 that fell into multiple categories. Up to three responses were categorized and combined for data analysis. Table 1 and Table 2 provide examples of the adopters’ written responses in each category for Questions 2 and 3. 

**Table 1:** Categories and typical responses for Question 2, “What was the single most important reason that you chose this particular pet?”

Response category	Actual response examples
Behavior with people	Seems sociable; friendly; the way he interacts with me
Personality/Temperament	Personality; temperament
Appearance	He is small; cute; he is fat; color; size; long haired; Yorkie
Bond/Love/Connection	We clicked; felt a connection to her; cat chose us
Playful	Playful, very playful
Energy level	Active; calm; mellow; liveliness
Sex	Wanted a male; female
Age	Looking for a kitten; looking for a puppy; feel sorry for older pets; looking for a young dog
Child/Family friendly	My son chose him; likes my kids; good with children
Similar to another pet	Reminded of a previous kitten; reminded me of my former cat
Companion for person(s) Behavior with animals/companion for pet	Seemed like a fun companion; companionship She will get along with our cat; compatible with our other dog; my other dog needs a friend
Special needs/pet really needing a home	Partially blind; she was special needs; she needs medical attention and could provide that for her.
Other	Easy to take care; perfect; security purposes; I love dogs

**Table 2:** Categories and typical responses for Question 3, “What did this pet do when you first met him/her?”

Response category	Actual response examples
Approach/Greet	Came right up to me; came over to investigate
Friendly/Snuggle/Affectionate	Very affectionate; cuddled in my arms
Licked	Licked me; licked my hands; kiss me
Jumped up/Climbed on	Jumped on me; climbed to my chest
Wagged	Wagged her tail
Looked at person	Made eye contact; looked at me
Moved around	Walked around; investigated surroundings
Shy/Avoid/Move away	Ran; shy
Allowed to pet	Let me pet her; responded to stroking; liked petting
Allowed to pick up/hold/sat in lap	Let me hold her; sat on my lap; melted in my arms
Played	Played with toys, played
Reached paw	She gave us her paw; reached her paw out
Rolled over	Rolled on his back
Rubbed on/Lean	Rubbing against my leg; rubbed against us
Sniffed	Smelled my hand, smelled us
Social behavior to animal	Played well with our dog; sniffing our pet
Stayed where he was	Stood up; nothing; sat still
Vocalized	Purred; meowed
Other	Woke up; urinated; did not hiss; swatted my finger

Frequencies were calculated for all categorical responses then Chi-square analysis was applied to examine potential differences between the data for cats and dogs, kittens and cats, and puppies and dogs. 

To correct for the varying line lengths in the printed visual analog rating scales (Questions 6 and 7 regarding the importance of seeing various aspects of the animals’ behavior and of various types of information provided about the pet, respectively), the answers were converted to percent of total line length. Neither the natural log nor square root normalized the kurtotic and/or skewed distributions of these ratings; therefore Friedman’s test was used to compare the relative importance of seeing the animal’s behavior in the three different contexts and of each information type. Then Mann-Whitney U tests were applied to compare species and age groups of animal. 

## 3. Results

A total of 1,599 adopters completed the survey. Of the completed surveys, 1,491 had sufficient information on species and age of adopted animal to complete analysis. Only one person declined to participate and one person completed the survey in Spanish.

**Figure 2:**
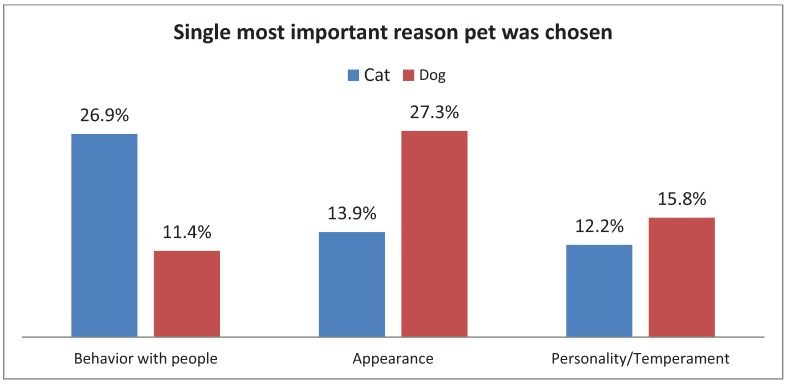
Adopter’s responses when asked the most important reason they choose their adult dog or cat.

**Figure 3:**
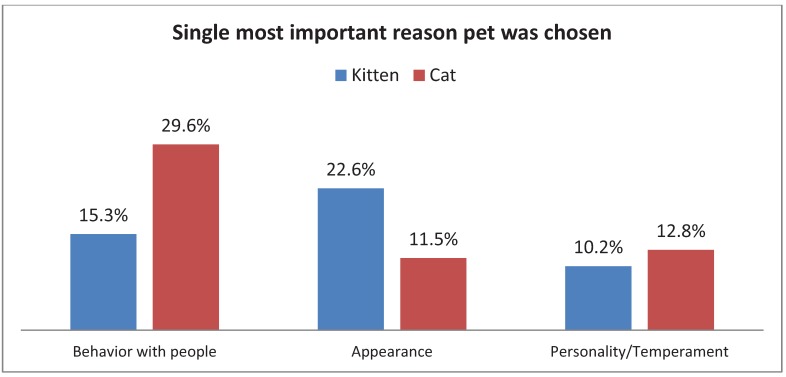
Adopter’s responses when asked the most important reason they choose their kitten or adult cat.

**Figure 4:**
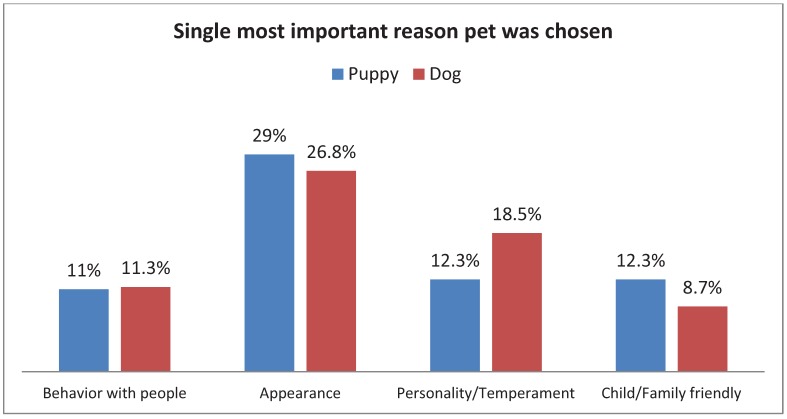
Adopter’s responses when asked the most important reason they choose their puppy or adult dog.

Overall, more dogs (54%) than cats (46%) were adopted. More puppies (69.4%) were adopted than kittens (30.6%), while slightly more adult cats (51.6%) were adopted than adult dogs (48.4%). Appearance was cited most often as the single most important reason people adopted their dog (27.3%) while 26.9% of people who adopted cats cited behavior (Figure 2). The above relationships between species and single most important reason for adoption were significant, χ^2^(1, N = 1,596) = 110.30, p < 0.005. Species type had a moderate relationship (Cramer’s V = 0.26) to most important reason cited. Within species, there was an effect of the animal’s reported age group (Figure 3), with appearance being the most frequently cited reason for adoption of a kitten (22.6%) while behavior with people was the most frequently cited reason for adoption of a cat (29.6%). In contrast, appearance was the most frequently cited reason (Figure 4), whether one was adopting a puppy (29%) or a dog (26.8%). For both cats and dogs, the relationship between the reported age group and the most important reason cited was significant, χ^2^(14, N = 675) = 34.38, p < 0.01, and χ2(14, N = 813) = 27.48, p < 0.05, respectively. Age group had a moderate relationship (V = 0.23) to single most important reason for cats, and a small effect (V = 0.18) for dogs.

Most people reported that the first thing their pet did when they first met him/her was approach or greet them (Cats = 19.8%, Dogs = 23%). However, the next frequently cited actions differed for cats and dogs (see Table 3). The relationship between species and pet’s first action is significant, χ^2^(18, N = 1,573) = 361.18, p < 0.005. The effect size is large (Cramer’s V = 0.48).

**Table 3:** Three most common responses to the question three: “What did this pet do when you first met him/her?”

All Cats	N	%
Approached/Greeted	143	19.8
Vocalized	97	13.4
Rubbed/Leaned on	67	9.3
**All Dogs**	**N**	**%**
Approached/Greeted	196	23
Licked	126	14.8
Jumped up/Climbed on	80	9.4

**Figure 5:**
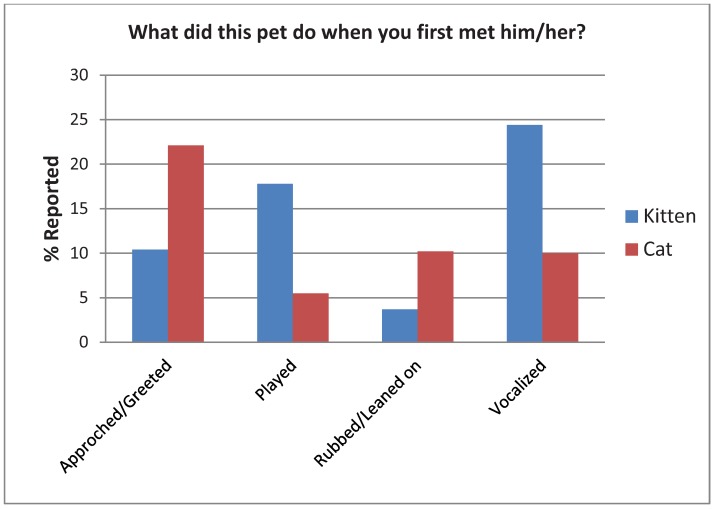
Adopter’s responses when asked the first behavior their kitten or cat exhibited upon meeting them.

Most people reported that the first thing their kitten did when they first met him/her was vocalize (33%) while most people reported their cat first approached or greeted them (22%) as seen in Figure 5. A chi-square suggests the differences in total responses between kittens and cats is significant, χ^2^(17) = 75.76, *p* < 0.005. The effect size is moderate (Cramer’s V = 0.34).

**Figure 6:**
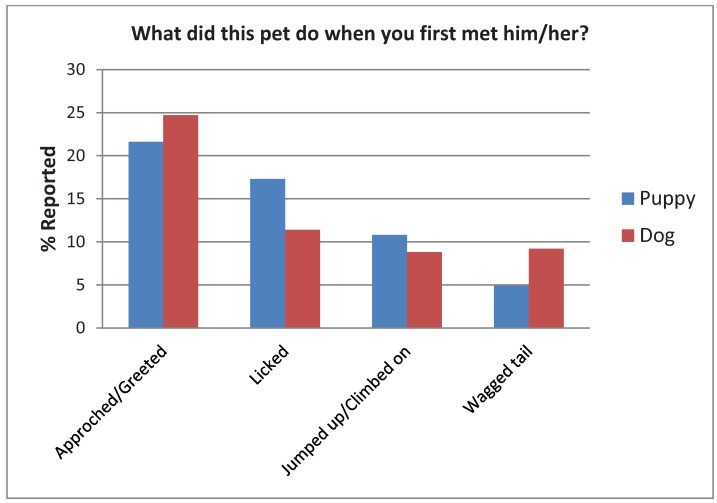
Adopter’s responses when asked the first behavior their puppy or dog exhibited upon meeting them.

Most people reported that the first thing their adopted canine did when they first met him/her was approach or greet them followed by licking (Figure 6). There was no significant difference between responses for puppies *vs.* dogs, χ^2^(18) = 24.23, *p* = 0.12.

When asked if any of the following reasons (listed in multiple choice formats) were important to their adoption selection, responses were very similar to those in the open-ended question asking the single most important reason for choosing that animal (Table 4). Appearance, behavior with people, and age were the most frequently selected reasons for all species and groups, whereas playfulness was among the top four reasons for kittens and puppies. 

**Table 4:** Responses to the question, “Were the following reasons important to your decision to choose this particular pet?”

**Reasons**	**Cats**				**Dogs**			
	Kitten		Adult		Puppy		Adult	
	N	%	N	%	N	%	N	%
Physical appearance	86	62.8	353	65.6	238	76.8	380	75.4
Age	107	78.1	344	63.9	232	74.8	330	65.6
Behavior with people	95	69.3	419	77.9	229	73.9	393	78.3
Playfulness	92	67.2	288	53.5	199	64.2	293	58.3
Sex	60	43.8	189	35.1	138	44.5	173	34.5
Health	69	50.4	276	51.3	139	44.8	247	49.1
Energy level	61	44.5	239	44.4	174	56.1	281	56
Behavior when by himself/herself	35	25.5	137	25.5	104	33.7	147	29.2
Behavior towards other animals	26	19	135	25.1	85	27.4	162	32.2
Recommendation from staff/volunteer	18	13.1	120	22.3	49	15.8	119	23.7
A special adoption offer	3	2.2	32	5.9	9	2.9	35	7
Wanted to help animal	18	13.1	113	21	70	22.6	119	23.7
Other	8	5.8	35	6.5	18	5.8	27	5.4

**Figure 7:**
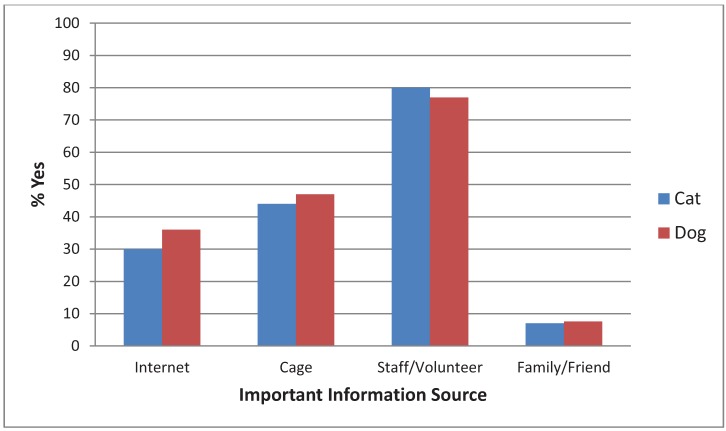
Adopter’s responses when asked which source provided the most important information about their dog or cat.

Most people reported that the most important source of information about their pet was from a staff member or volunteer (Figure 7). There was no significant difference between cats and dogs. More people reported that information from the internet was an important information source about dogs (36%) than cats (30%), (χ^2^(1, N = 1594) = 6.58, *p* < 0.01), however, the effect size was extremely small (Phi = 0.06). There was no significant difference in importance of information sources for cats *vs.* kittens. More dog adopters than puppy adopters reported that information from a staff member was important (36%), (χ^2^(1, N = 812) = 6.21, *p* < 0.02), but the effect size was very small (Phi = 0.09).

For both cats and dogs, seeing the pet's behavior when interacting with them was more important (M rank = 2.4) than seeing the pet behind the cage door (M rank = 1.84) or seeing the pet's behavior towards other animals (M rank = 1.76), χ^2^(2, N = 1,106) = 321.16, *p* < 0.001.

**Figure 8:**
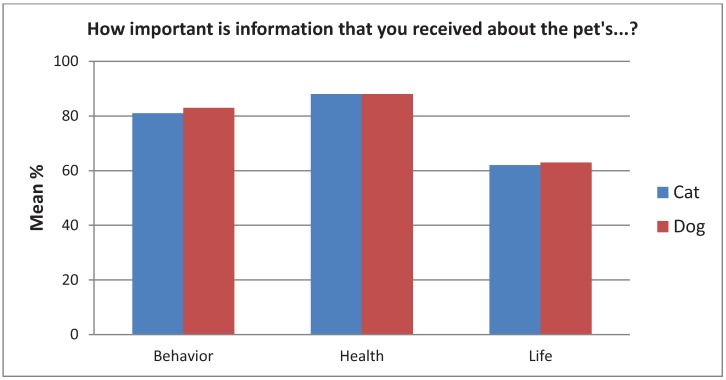
The importance of behavior, health, or prior life experiences of the dog or cat.

Figure 8 illustrates that receiving information about the pet's health (M rank = 2.37) was more important than receiving information about the pet's behavior (M rank = 2.06) or about the pet's life before entering the shelter (M rank = 1.57), χ^2^(2, N = 1,441) = 575.41, *p* < 0.001.

Receiving information about dogs’ behavior (M rank = 802.53) was more important than receiving information about cats’ behavior (M rank = 751.43), Z = −2.24, *p* = 0.03. There was no significant difference in how important the information was about cats’ *vs.* dogs’ health or previous life. There were no differences in importance of information with regards to cats *vs.* kittens, whereas receiving information about dogs’ behavior (M rank = 419.55) was more important than receiving info about puppies’ behavior (M rank = 359.3), Z = −3.62, *p* < 0.001.

## 4. Discussion

This study examined the reasons adopters gave for their choice of animal, which animal behaviors were first exhibited by the pet and therefore may have played a role in the selection process, what avenues of information gathering were important during the selection process, the relative importance of seeing the animals’ behavior in various contexts, and the relative importance of various types of information about the animal. 

Similar to results of previous studies, appearance was cited among the top three reasons for choice of animal. Appearance was more important to dog adopters than cat adopters. This may be due to the greater diversity in dog breeds, body shapes, sizes, and hair types than among cats. Appearance was more frequently cited as a reason for adoption of a kitten than a cat. Adopters of adult cats were most likely to cite how the cat behaves towards people. Perhaps there is more variation in appearance among kittens, or the ‘cuteness factor’ is more obvious among kittens than cats, thereby attracting adopters’ attention. Programs like the ASPCA’s Meet Your Match Feline-ality program may be utilized to help guide adopters interested in adult cats to cats whose behavior best meets their needs and interests. Also providing a play toy at the front of the cage for adopters to engage the cat or training cats to come to the front of the cage can be utilized to highlight behavior traits of the cats.

Special adoption offers were infrequently selected as a factor important to the adoption selection. Special offers may encourage adoption in general, but the present findings suggest they are relatively unimportant to the individual choice of animal.

While this survey did not ask which of the pet’s behaviors were most important to the adoption choice, the fact that selected cats and dogs were both most likely to approach or greet the adopter when first met suggests the potential importance of social greeting behavior in catching attention or initiating a bond between human and animal. This finding is also in agreement with two previous research findings that dogs at the front of their kennels were preferred by potential adopters over those at the back [10] and that time to adoption of shelter cats is positively correlated with latency to approach an unfamiliar person [14].

Shelters may wish to encourage approach behaviors by teaching animals to come the front of the kennel when people pass by, for example by asking staff and volunteers to provide a treat from a cup hung on the kennel door. This training would also provide positive association with people among fearful animals, so that they are more likely to offer the other pro-social behaviors most commonly cited in this survey, such as rubbing on or leaning on people among cats, and licking and approaching among dogs.

It is interesting that jumping up was the third most common behavior dogs exhibited when first met. While the present results do not indicate whether adopters actually liked this behavior, it is possible that they found this to be a friendly and bond-initiating behavior that positively affected their adoption decision. Further study is clearly needed into which animal behaviors are most important to adopters so that shelters can make informed decisions about which to encourage and which to discourage.

Regardless of whether they adopted a cat or dog, most people reported that an important source of information about their pet was from a staff member or volunteer. In an age where shelters are spending more time and energy on developing their websites and cutting staff to save money, this study indicates that adopters find no substitute for a person helping them select an animal. Staff members and volunteers can guide adopters toward animals that best fit the adopter’s lifestyle and living situation, so that adoptions can be informed by more than just appearance. A word of caution should be noted as well, as the information provided by the staff member or volunteer about the pet should be accurate, as sending adopters home with incorrect information about what to expect regarding their new pet could put the bond at risk [17]. 

Interacting directly with the dog or cat was rated across species as more important than seeing the pet behind a cage door. This was similar to previous research on environmental influences on cat adoption [12]. Up-close, hands-on interaction may be most important for animals that have been repeatedly passed over by adopters. Shelters should consider taking these animals among visiting adopters or among the general public outside of the shelter in order to provide such hands-on interaction that adopters find so important. Dogs can be taken out of their kennel with a no-pull harness and cats can be trained to accept a harness in order to provide them these opportunities. This may be even more important for dogs that do not show highly adoptable behavior while behind a kennel door.

Receiving information about the pet’s health was more important than the pet's behavior or information about the pet's life before entering the shelter. We do not know how respondents interpreted this question or how much information they actually received from the shelter on these topics. Almost all animals would have some health and behavioral information available because these could be observed by shelter staff during the animal’s stay. However, many animals may have little or no information available on their former life.

These present results provide some insight into ways to reduce returns of adopted animals. For example, it appears that adopters are often choosing puppies, dogs, and kittens based mainly on appearance. Therefore, when interacting with potential adopters, shelter staff could emphasis behavior and personality to create a better match. Furthermore, even though adopters seem to place relatively low priority on seeing an animal’s behavior with other animals, staff might discuss how to introduce the new pet and build a positive inter-animal relationship because most owned dogs and cats in the US live in a household with at least one other member of their species [18].

Aside from better adoption matches, shelters might also use the present findings to prioritize resources. For example, knowing that adopters find information from staff and volunteers more important than cage card information, shelters might allocate their resources to focus more on face-to-face interactions with adopters. Also, since health and behavior information are more important than information on an animal’s former life, the time spent with adopters could be economized by focusing discussion on these topics. Lastly, since adopters are most swayed by the time they spend interacting directly with animals, more resources could be spent ensuring the public can interact with the animals in a variety of ways. For example, shelters could provide more visiting rooms, longer visiting times, play runs, and options to interact with the animals while they are waiting, such as a treat cup or a toy to interact with the dog or cat through the cage door when possible.

## 5. Conclusions

Overall, the appearance of the animal, social behavior with adopter, and personality were the top reasons for adoption across all species and groups. Most adopters obtain the information important for adoption selections by interacting with a staff member and directly with the animal. The results of this study can be used to create better adoptions and prioritize shelter resources to increase adoptions. 
